# Applications of Lasers in Bioanalytical Chemistry

**DOI:** 10.6028/jres.093.136

**Published:** 1988-06-01

**Authors:** Edward S. Yeung

**Affiliations:** Department of Chemistry, Iowa State University, Ames, IA 50011

The combination of laser detectors and liquid chromatography (LC) has led to new types of analytical measurements in solution. LC has benefited from these selective detectors because, for complex biological matrices, complete separation of the analytes is rarely possible. Examples are polarimetry for distinguishing biologically important species from the bulk biological fluids and two-photon excited fluorescence. Laser spectroscopy has also benefited from the sample clean-up step provided by the LC, and the fact that the LC baseline allows a convenient reference measurement.

Absorption is a difficult spectroscopic measurement in remote monitoring situations. A well defined pathlength must be present to avoid nonlinear response. Unlike fluorescence, the measured radiation is at the same wavelength as the incident radiation, and scattered light is a problem. It is possible to use two optical fibers to direct light in and then out of the sample area, at the expense of a larger probe volume and potential cross-talk between fibers. When one fiber is used, reflections and scattering from the optics must be overcome. We use index-matching fluids to compensate for reflections at the end faces of the fiber. By modulating the laser source at a high frequency, the phase difference of the signals at the entrance of the fiber and at the remote optical region can be adjusted to 90°. Then, a lock-in amplifier can be used to distinguish the two, leading to accurate absorbance values (figs. 1 and 2). We used this system for remote absorption detection of the effluent from a microbore LC column, and good detection capabilities were obtained.

In the last decade, the use of high performance liquid chromatography (HPLC) for protein separation and purification has increased dramatically. Reversed-phase (RP) separations are popular because of the high resolving power available and widespread use of RP-HPLC in many other areas. But the conditions which produce good separations are known to significantly change the protein structure. Broadened or multiple peaks resulting from subjecting a single protein to RP-HPLC have been observed by several researchers for a variety of proteins. The HPLC of soybean trypsin inhibitor was reexamined by using simultaneous optical activity and ultraviolet absorption detection (fig. 3). Ratio plots of the two detector responses allow easy identification of impurities that were not related to the protein (fig. 4). The specific rotations of each of the separated components can be derived. We find that one denatured form has a distinctly lower specific rotation while another form shows no change in specific rotation. The on-column denaturation rate here was found to be slower than that from previous work. Column pretreatment may have resulted in milder column conditions through the elimination of irreversibly adsorbing sites.

## Figures and Tables

**Figure 1:**
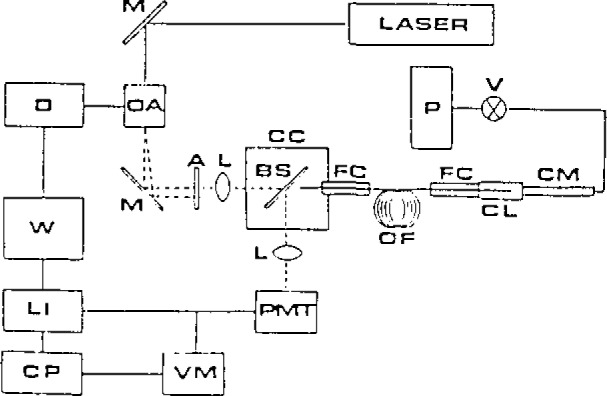
Fiber optic absorbance probe and chromatographic system: P, pump; V, injection valve; CM, microbore column; CL, absorbance cell; FC, fiber chuck; OF, optical fiber; CC, coupling cell; BS, beam splitter; L, lens; A, aperture; M, mirror; OA, Bragg cell; Laser, HeNe laser; D, driver; W, square-wave generator; LI, lock-in amplifier; CP, computer; VM voltmeter; PMT, photomultiplier tube.

**Figure 2:**
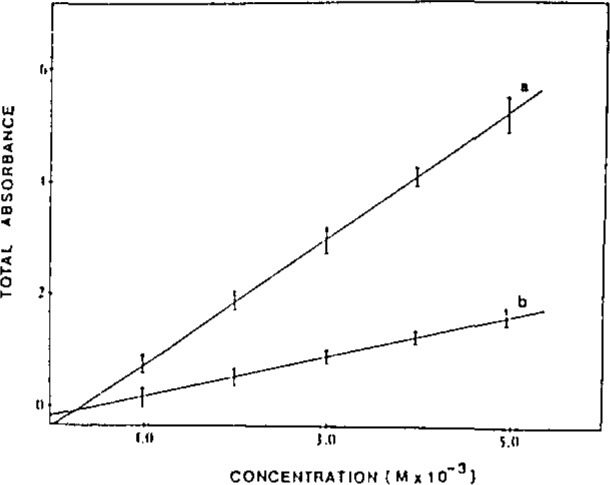
Beer’s law plots of (a) modulated and (b) nonmodulated bromcresol green absorbance signals. Plots represent 95% confidence intervals for three replicate measurements.

**Figure 3:**
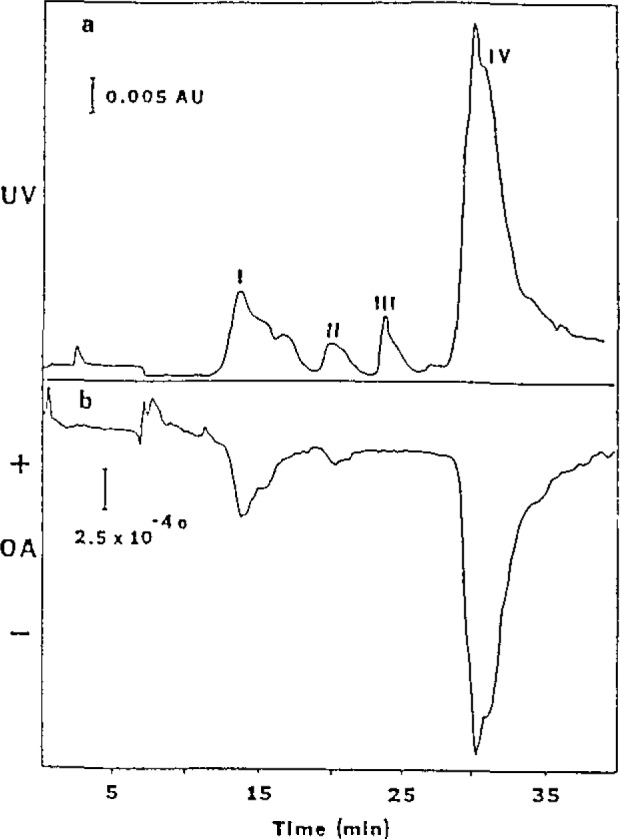
Ultraviolet absorbance (a) and optical activity (b) chromatograms of soybean trypsin inhibitor, 115 μg injected.

**Figure 4:**
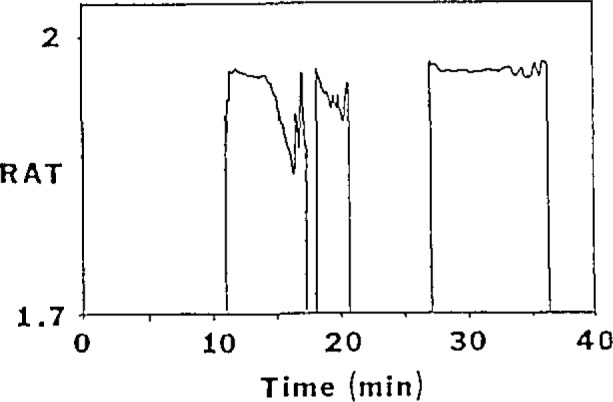
Plot of ratio (RAT) values versus time for the chromatograms shown in figure 3.

